# Changes in physical activity during the transition from primary to secondary school in Belgian children: what is the role of the school environment?

**DOI:** 10.1186/1471-2458-14-261

**Published:** 2014-03-19

**Authors:** Femke De Meester, Delfien Van Dyck, Ilse De Bourdeaudhuij, Benedicte Deforche, Greet Cardon

**Affiliations:** 1 Department of Movement and Sport Sciences, Faculty of Medicine and Health Sciences, Ghent University, Brussels, Belgium; 2 Research Foundation Flanders (FWO), Brussels, Belgium; 3 Department of Human Biometry and Biomechanics, Faculty of Physical Education and Physiotherapy, Vrije Universiteit, Brussels, Belgium

## Abstract

**Background:**

Key life periods have been associated with changes in physical activity (PA). This study investigated (1) how PA changes when primary school children transfer to secondary school, (2) if school environmental characteristics differ between primary and secondary schools and (3) if changes in school environmental characteristics can predict changes in PA in Belgian schoolchildren. Moderating effects of gender and the baseline level of PA were investigated for the first and third research question.

**Methods:**

In total, 736 children (10–13 years) of the last year of primary school participated in the first phase of this longitudinal study. Two years later, 502 of these children (68.2%) agreed to participate in the second phase. Accelerometers, pedometers and the Flemish Physical Activity Questionnaire were used to measure PA. School environmental characteristics were reported by the school principals. Cross-classified regression models were conducted to analyze the data.

**Results:**

***S*** elf-reported active transport to school and accelerometer weekday moderate to vigorous PA (MVPA) increased after the transition to secondary school while self-reported extracurricular PA and total PA decreased. Pedometer weekday step counts decreased, but this decrease was only apparent among those who achieved the PA guidelines in primary school.

Secondary schools scored higher on the school environmental characteristics: provision of sports and PA during lunch break, active schoolyards and playgrounds and health education policy but lower on sports and PA after-school than primary schools.

Changes in the school environmental characteristics: active commuting to school, active schoolyards and playgrounds and health education policy resulted in changes in self-reported extracurricular PA, total PA , pedometer/accelerometer determined step counts and accelerometer determined MVPA. Moderating effects were found for baseline PA and gender.

**Conclusion:**

PA changed after the transition to secondary school. In general, secondary schools seem more likely to foster strategies to promote PA during school hours than primary schools who seem more likely to foster strategies to promote PA after school. Changes in school environmental characteristics may contribute to changes in PA. Thus, if confirmed in future studies, efforts are needed to implement these components in schools as early as possible to positively affect the change in PA.

## Background

The promotion of physical activity (PA) among young people has become a public health priority because of the well-known health benefits [1,2]. The belief that the behavior of young people is easier to control, to develop or to change into more healthy behavior than the behavior of adults [3], underscores the importance of primary prevention in young people. Furthermore, persistent PA at a young age considerably increases the probability of being active in adulthood [4] which in turn can contribute to the quality of adult life. Despite these benefits, a large proportion of school-aged youth does not achieve the public health recommendations of 60 minutes moderate-to-vigorous physical activity (MVPA) per day [5]. Moreover, PA levels tend to decline during adolescence [6,7].

Research has shown that important life events are key periods that coincide with changes in PA patterns [8]. For children, the transition from primary to secondary school is an important life event. Many European countries including Belgium have distinct primary and secondary schools. In the Belgian educational context, children spend the first 9 years in a primary school (3–11 year olds) after which they normally move to a secondary school (mostly located elsewhere) where they spend 6 years (12–18 year olds). Different longitudinal studies have shown that the transition from primary to secondary school is characterized by changes in PA patterns has been shown by different longitudinal studies [8-12]. However, the directions of these changes are not straightforward. Two longitudinal studies have shown that PA levels gradually decreased during childhood but were characterized by an obvious drop during the transition from primary to secondary school [8,9]. Specifically, Niven and colleagues observed a decrease in total PA, PA during school day break and lunch time and PA after school. In contrast, Cooper and colleagues described that objectively determined MVPA slightly increased during this transition [10]. Jago and colleagues [11] investigated after-school and weekend MVPA and concluded that objectively determined after-school MVPA tended to decline during the transition from primary to secondary school, while objectively determined weekend MVPA tended to increase during this transition. Furthermore, Cardon and colleagues found an increase in time spent bicycling to school during the transition from primary to secondary school [12]. Based on these results, the changes in PA during the transition from primary to secondary school seem to be dependent on the context of PA. Furthermore, according to several authors the changes seem to be more apparent in boys than in girls [9,11]. The results of these longitudinal studies clearly underscore the need for studies to further scrutinize the change in context-specific PA during the transition from primary to secondary school.

An important question to inform the development of PA interventions is why PA changes during this transition. In line with the perspectives of the ecological models, behavior change occurs as a result of the dynamic interaction between the individual and the environment in which he/she lives, learns, works and plays [13]. Young people spend a large proportion of their time at school. Virtually all children and adolescents attend school-based education. In general, children and adolescents spend between 4 and 8 hours per day for 12 years or more of their lives at school. Schools therefore have the opportunity to offer an environment with the potential to facilitate and to promote PA [14] in a large number of children and adolescents, across different ethnic and socio-economic groups. To foster regular PA, schools worldwide are recommended to strive for a ‘whole-school’ approach that maximizes the opportunities for school-aged youth to be active [15,16]. The ‘whole-school’ approach that has been outlined in the Toronto Charter for Physical Activity [17] goes beyond providing physical education classes. Key components of a ‘whole-school’ approach are: providing resources and suitable physical environments to support structured as well as unstructured PA, supporting active travel to school and provision of opportunities to be active during the school day, during class, during breaks at lunch time and after school (Toronto charter). In line with the ‘whole-school’ approach, a conceptual framework for PA programs within school-community partnerships was developed for the schools in Flanders (Belgium) to offer schools guidelines to develop extracurricular PA programs [18]. The framework consists of five complementary components: sports and PA during lunch break, active school yards or playgrounds, active commuting to school, health education policy and sports and PA after school. Since a few years, Flemish primary and secondary schools are sensitized and supported to have a school policy that promotes PA. However, no specific strategies were developed by the Flemish government: the Flemish schools are free to choose which promotion programs they implement. Consequently, the extent of PA promotion differs; some schools focus on one of the five framework components, while other schools focus on all five framework components [19]. The differences in approaches between schools may contribute to the change in PA observed when moving schools.

To our knowledge, the only study, investigating the contribution of school environmental factors to the change in PA when moving schools was a qualitative study [20]. Through the use of narrative interviews, Knowles and colleagues (2011) investigated factors (PA opportunities, environment, sense of self when active and individual issues) related to the decrease in PA during the transition between primary and secondary school in 14 British girls. A main conclusion of this study was that the change in school environment plays a central role in the change in PA patterns that occur when moving schools [20]. Specifically, according to this study, a positive environment could be provided by ensuring a choice of activities in the physical education lessons, reducing the focus on competence and competition and recognizing the importance of social support by the involvement of friends in PA [20].

Based on the shortcomings in the present literature, the aim of this study was threefold.

A first study aim was to investigate how PA behavior changes when Belgian primary school children make the transition to secondary school. Changes in self-reported walking and cycling to and from school, extracurricular activity at school and total PA as well as changes in pedometer and accelerometer determined step counts and MVPA were examined. A second aim was to investigate if school environmental characteristics with regard to the five framework components (sports and PA during lunch break, active school yards or playgrounds, active commuting to school, health education policy and sports and PA after school) differed between Belgian primary and secondary schools. The third and last aim of this study was to investigate if changes in school environmental characteristics can predict changes in PA in Belgian schoolchildren. Since in previous studies the changes in PA during the transition from primary to secondary school were found to be more apparent in boys than in girls [9,11], the moderating effects of gender was investigated with regard to the first and third research question. Furthermore, since change in PA over time has shown to be related to the start level of PA [21], the moderating effects of the level of PA at baseline was also investigated with regard to the first and third research question.

## Methods

### Participants and procedure

A longitudinal study with two phases was conducted: baseline measurements took place during the school year 2009–2010, follow up measurements took place during the school year 2011–2012.

During the school year 2009–2010, 148 schools were randomly selected from all elementary schools in East- and West-Flanders. The principals of these schools were contacted by phone and informed about the study. Forty-four principals agreed to participate in the study (response rate at school level = 29.7%) and were visited during school hours. They were asked to give written consent and filled in a questionnaire on school environmental characteristics.

Further, with the permission of the principal, the children of one class group of the final year (10–13 year old) and their parents were invited to participate in the study. In total, 976 children and parents received an informative letter about the study with an invitation to participate. The parents of 749 children consented to be involved in the study and agreed to let their child participate in the study (response rate at individual level = 76.7%). Each class that participated in the study was visited by a research assistant. During this visit, the children involved in the study and present at the time of the visit (n = 736, 98.3%) were asked to complete a questionnaire. The questionnaires were completed under the supervision of the research assistant.

Every child was also asked to wear an activity monitor for seven consecutive days. In total, 439 (59.6%) children received a pedometer measuring step counts (Yamax Digi-walker CW-701) and because of the limited availability of accelerometers a subsample of 297 (40.4%) children received an accelerometer measuring activity counts per minute and step counts (model GT1M, Actigraph MTI, Manufacturing Technology Inc., Pensacola, FL, USA). The activity monitor was accompanied by a non-wear time activity diary. The research assistant explained the protocol of the activity monitor and its non-wear time activity diary. At the end of the visit, every child was given a questionnaire to be completed by one of the parents at home.

One week later, the research assistant revisited the schools to collect the parental questionnaires, the activity monitors and the non-wear time activity diaries. The parental questionnaires, activity monitors or diaries that were forgotten at that time, were either redirected by post or collected by the research assistant during a third visit.

After the first phase of the study, 736 (98.3%) child questionnaires were received, 686 (93.2%) children had complete pedometer or accelerometer step count data for weekdays and 273 (91.9%) children had complete accelerometer data (activity counts per minute) to determine weekday MVPA. Furthermore, from 93.5% of the parents (n = 701), a complete parent questionnaire was returned to school and from all 44 schools we received the completed questionnaire concerning the school environmental characteristics.

During the school year 2011–2012, follow up measurements took place. Since the children had moved schools, they could not be contacted through the school. Therefore, children and parents (n = 736) who participated in the first phase of the study were contacted by phone to ask if they were willing to participate in the second phase. In total, 502 (68.2%) children and their parents agreed to participate in the second phase of the study. Eighty-seven children (11.8%) declined to participate and 147 (20.0%) were not reached after three attempts on different days and times of the day.

The children who consented to participate, who were willing to wear an activity monitor (n = 427) and had worn a pedometer during the first phase (n = 249) received an envelope via regular mail, containing a pedometer, a non-wear time activity diary, a child questionnaire, a parent questionnaire and a manual with guidelines concerning the pedometer, the non-wear time activity diary and the questionnaires. The envelope also contained a pre-stamped envelope to send everything back via regular mail.

The children who consented to participate, who were willing to wear an activity monitor (n = 427) and had worn an accelerometer during the first phase (n = 178) were visited at home. During the home visit, the accelerometer, the non-wear time activity diary, the child questionnaire, the parental questionnaire and the manual were delivered. One week later, during a second home visit, everything was collected.

The principals of the 124 secondary schools, in which the children entered after primary school, were contacted by phone and asked to fill in a questionnaire on school environmental characteristics. In total, 107 (86.3%) schools were willing to participate, 17 (13.7%) declined to participate. The questionnaires were sent to the schools via regular mail. The envelope contained a pre-stamped envelope to send the questionnaire back. The study protocol received approval from the Ethics Committee of Ghent University Hospital.

At the end of the second phase of the study, 420 (83.6%) child questionnaires returned, 369 (86.4%) children had complete pedometer or accelerometer step count data for weekdays and 140 (78.7%) children had complete accelerometer data for weekdays. Furthermore, we received 416 (82.8%) parent questionnaires and 100 (93.5%) schools returned the school questionnaire.

### Measures

The outcome and exposure measures that were of interest for the purposes of this study are described in the following sections. The way in which these measures were obtained, was identical at baseline and follow up.

### Demographic factors

In the child questionnaire, the child’s age and gender were assessed. The parent questionnaire contained questions about their own and their partner’s level of education. The educational attainment of the children’s parents was used as a proxy measure of children’s socio-economic status. The educational level of the child’s mother and father was determined based on four options: less than high school, completed high school, completed college or completed university. The educational levels of mother and father were coded into ‘reached a college or a university education level’ or ‘did not reach a college or a university education level’.

### School environmental characteristics

A school questionnaire was developed and used to assess the implementation of the five framework components concerning extracurricular PA promotion on schools: active commuting to school, sports and PA after school, sports and PA during lunch break, active school yards or playgrounds and health education policy. An outline of the content and response options of the 24 items is given in Table 1.

**Table 1:** Content and response options of the different items included in the school environmental questionnaire

**Framework components and associated themes**	**Content of items**	**Response**
***Active commuting to school***		
*Promotion of active school commuting:*	- Active school commuting is promoted by your school.	5-point scale^a^
*Facilities for active school commuting:*	- There are sufficient bicycle racks on school for the students.	5-point scale^a^
	- There are cycle lanes and footpaths in the school neighborhood.	5-point scale^a^
	- The roads in the school neighborhood are well lit.	5-point scale^a^
*Safety from traffic:*	- There is a lot of traffic in the school neighborhood	5-point scale^a^
	- There are dangerous crossings in the school neighborhood	5-point scale^a^
*Safety from theft:*	- The bicycle racks on school are safe	5-point scale^a^
***Sports and physical activity after school:***		
*Physical activity after school hours:*	- Does the school organize sports and physical activity after school (before or after school hours or on Wednesday afternoon)?	Binary^b^
*Use of facilities:*	- Are the students allowed to use school facilities (e.g. sports hall, polyvalent spaces, covered play areas, fields of grass, outdoor sports fields) after school hours?	Binary^b^
*Use of equipment:*	- Are the students allowed to use school sports equipment (e.g. small sports and play material, loan desk for material, music installation, lockers, lines, goals en nets) after school hours?	Binary^b^
*Promotion of after school activities:*	- The school promotes sports and physical activity after school.	5-point scale^a^
*Information about activities after school:*	- The school provides information about the sports and physical activity possibilities in the village/city/town	5-point scale^a^
*Cooperation with local partners:*	- Organizations, sports clubs and other local initiatives can use the school facilities after school hours.	5-point scale^a^
	- The school cooperates with community partners e.g. there is cooperation with local sports clubs.	5-point scale^a^
***Sports and physical activity during lunch break***		
*Physical activity during lunch break:*	- Does the school organize after sports and physical activity during lunch break?	Binary^b^
*Promotion of physical activity during lunch break:*	- The school stimulates the students to use the school sports facilities and equipment during the school hours.	5-point scale^a^
	- The school stimulates the teachers to participate in the sports and physical activities during lunch break and recess.	5-point scale^a^
	- During recess the teachers stimulate the students to be active (e.g. by motivating the students, by participating)	5-point scale^a^
	- During recess the teachers participate in the sports and physical activities.	5-point scale^a^
***Active schoolyards or playgrounds***		
*Available of facilities*	- Which school facilities (e.g. sports hall, polyvalent spaces, covered play areas, fields of grass, outdoor sports fields) can the students use during recess and lunch break?	continuous
*Available of equipment*	- Which sports equipment (e.g. small sports and play material, loan desk for material, music installation, lockers, lines, goals en nets) can the after school hours?	continuous
***Health education policy***		
*Subjective norm:*	- How important is sport and physical activity for the school?	10-point scale
*Involvement of students:*	- Are pupils involved in decision making about sport and physical activity?	5-point scale^a^
*Training for teachers:*	- The school supports teachers to have training on sports and physical activity	5-point scale^a^

^a^Strongly disagree, somewhat disagree, neither agree or disagree, somewhat agree, strongly agree.

^b^yes, no.

Active commuting to school was questioned by seven items on four themes: promotion of active school commuting, facilities for active school commuting, safety from traffic and safety from theft. Sports and PA after school was questioned by seven items on six themes: PA after school hours, use of facilities, use of equipment, promotion of activities after school, information about activities after school and cooperation with local partners. Sports and PA during lunch break was questioned by five items on 2 themes: PA during lunch break and promotion of PA during lunch break. Active school yards or playgrounds was questioned by two items on two themes: availability of facilities and availability of equipment. Health education policy was questioned by three items on three themes: subjective norm, involvement of students and training of teachers. The questions were mainly based on previous research investigating the PA promotion framework in Flemish elementary and secondary school children [19] and on the questionnaire used in the New South Wales (NSW) Schools Physical Activity and Nutrition Survey (SPANS) [22].

### Physical activity

#### Self-reported physical activity

To determine the duration (hours and minutes per day) of school related active transportation (walking and cycling to and from school), extracurricular activity at school (participation in physical activities during playtime, lunch break, after school hours or at class or school tournaments) and total PA (including school related active transportation, leisure time active transportation, physical education, extracurricular PA at school and sports during leisure time), the Flemish Physical Activity Questionnaire (FPAQ) was used [23]. The paper and pencil version of the FPAQ was found to be a reliable and reasonably valid questionnaire for the assessment of different dimensions of PA in children, especially when completed with (parental) assistance (test-retest reliability coefficients: ICC = 0.74 to 0.93, with exception from ICC = 0.26 for leisure time active transportation and criterion validity: r = 0.27-0.44) [24], and in adolescents (test-retest reliability coefficients: ICC = 0.68 - 0.84 and criterion validity: r = 0.43-0.48, excepts for the extracurricular activity at school: r = −0.16 and school related active transportation: r = −0.19) [23].

#### Pedometer and accelerometer assessed physical activity

##### Weekday step counts and weekday moderate to vigorous physical activity

To measure weekday step counts, the Yamax Digiwalker SW-200 (Yamax cooperation, Tokio, Japan) and the Actigraph accelerometer, model GT1M (Actigraph MTI, Manufacturing Technology Inc., Pensacola, FL, USA) were used. The Yamax Digiwalker has been acknowledged as a valid, accurate and reliable pedometer to measure free-living step-counts in children [25]. The GT1M accelerometer has demonstrated good reliability for measuring steps [26]. Evidence exists that neither accelerometers nor pedometers are affected by reactivity among adolescents [27,28]. Although the step counts measured by the Yamax Digi-walker CW-701 (the update of the Yamax Digiwalker SW-200) have been shown to be highly correlated with the step counts of the GT1M accelerometer (r = 0.78), the overall agreement between the step counts of both monitors is rather low [29]. In the study of Kinnunen et al. (2011), the 95% limits of agreement ranged between −2690 to 2656 steps/day for the mean value (mean of accelerometer and pedometer steps/day = 6026). Further, the limits of agreement varied substantially over the range of values. At the lowest recorded step count (mean of accelerometer and pedometer steps/day = 906) the accelerometer was on average recording more steps/day than the pedometer. In contrast, at the highest step count value (mean of accelerometer and pedometer steps/day = 12,018) the accelerometer recorded less steps/day than the pedometer on average [29]. To overcome this problem, all analyses were controlled for the type of monitor used.

In the subsample that was asked to wear an Actigraph accelerometer instead of a pedometer (first phase n = 297, second phase n = 178), model GT1M (Actigraph MTI, Manufacturing Technology Inc., Pensacola, FL, USA) the monitor was also used to measure weekday MVPA. Actigraph accelerometers have shown to have good reproducibility, validity and feasibility in adolescents [30,31].

##### Protocol data reduction pedometer and accelerometer

The children were asked to wear the activity monitor (pedometer or accelerometer) for seven consecutive days including two weekend days. They were asked to wear the activity monitor during waking hours but to remove the activity monitor for aquatic activities and for activities that prohibit activity monitors. Together with their activity monitor, all children received a diary. These diaries were provided to register activities for which the activity monitor was removed. Adolescents recorded on the diaries when the activity monitor was removed, when they put it back on, and the kind of the activities they were involved in (e.g. swimming, football, gymnastics).

The non-wear activities described in the diaries were classified as sedentary (SED), light (LPA), moderate (MPA) or vigorous (VPA) PA according to their MET (Metabolic Equivalent Task) values estimated from the Compendium of energy Expenditure for Youth (SED <1.5 MET; LPA <3MET, MPA, 3–6 MET, VPA ≥6 MET) [32].

Children who wore a pedometer were also asked to record the date and steps taken at the end of the day in the diary. For every minute of reported MVPA registered in the diary for which the pedometer was removed, 150 steps were added to the daily number of reported step counts [33].

The accelerometer data were downloaded to a computer (Actilife software version 4.1.0). Data-reduction software, MeterPlus [34], was used to screen, clean and score the accelerometer-data (step counts and activity counts/min).

The data-reduction process of the step counts obtained by accelerometers was comparable to the data-reduction process of the step count data obtained by pedometers. For every minute of reported MVPA in the diary for which the accelerometer was removed 150 steps were added to the daily number of registered step counts [33].

In the data reduction process of the activity counts, time periods of at least one hour of consecutive zeros were removed assuming the accelerometer was unworn [35,36]. Whenever applicable, the activity diaries were used to replace these consecutive number of zeros by the corrected number of minutes moderate PA (MPA) and vigorous PA (VPA) registered in the diaries [37]. To score the accelerometer-data and to obtain the mean number of minutes/day MVPA, the cut-points of Puyau (MVPA: ≥3200 activity counts/min) [38] were used. The review of Reilly and colleagues (2008) concluded that current evidence suggests a cut-point within the range of 3000–3600 counts/min to determine MVPA when using the Actigraph with 1 minute epochs [39]. Puyau and colleagues defined MVPA as activity counts above 3200 counts/min. which lies in the range of 3000–3600 counts/min [38].

For inclusion in the data analysis, the required total accumulated number of minutes registered time by the accelerometer and diaries was 600 minutes/day.

For both activity monitors, pedometers and accelerometers, a last step in the data reduction process was the determination of a weekday average. A minimum of three valid weekdays of monitoring was needed to obtain reliable estimates [40,41]. The participants failing to reach the postulated inclusion criteria were excluded from the analyses. In total, 24 children (8%) were excluded from the analyses the first phase of the study, whereas 38 children (21%) were excluded from the analyses in the second phase of the study.

##### Data analyses

Descriptive statistical analyses were conducted using SPSS 20.0.

The outcome and exposure measures obtained for the purposes of this study originate from three levels: individual level, primary school level and secondary school level. These three levels do not fit in a hierarchical model that assumes a hierarchical or nested structure. Children from a certain primary school may attend several different secondary schools. This is a typical cross-classified structure [42]. The maximum likelihood procedures (for example IGLS algorithm) are designed to work well for nested structures but are not able to take into account a cross-classified structure. Therefore, the Markov Chain Monte Carlo Method (MCMC) was used to fit the cross-classified multilevel models [42]. This method treats each set of classification units as an additive term in the model [43].

To investigate longitudinal changes (primary-secondary school) in PA (self-reported PA: active transport to and from school, extracurricular PA at school and total PA; pedometer/accelerometer determined weekday step counts and accelerometer determined MVPA) and differences in the implementation scores of the five framework components concerning extracurricular PA promotion in schools (active commuting to school, sports and PA after school, sports and PA during lunch break, active school yards or playgrounds and health education policy), four-level (time point, individual, primary school, secondary school) cross-classified multilevel regression models were conducted using MLwin version 2.22. Longitudinal changes were investigated by regressing the dependent PA variables and the implementation scores of the five framework components onto the time point variable. To investigate if longitudinal changes in PA are moderated by gender or by the level of PA in primary school, interaction effects with gender and the level of PA in primary school (expressed in ‘achieved the guidelines or not’) were examined by entering the cross-product term “time point × gender” and “time point × baseline PA” in the regression models. Girls achieved the guidelines when their pedometer/accelerometer determined mean number of steps on weekdays was higher than 11.000, boys achieved the guidelines when their mean number of steps on weekdays was higher than 13.000 [44].

To investigate if the changes in the implementation scores of the five framework components concerning extracurricular PA promotion on schools are predictors of changes in PA, cross-classified multilevel regression models were conducted. The changes in the implementation scores of the five framework components were regressed onto the changes in PA. To investigate if the association between the five framework components and PA is moderated by gender or by the level of PA in primary school, interaction effects with gender and the level of PA in primary school (expressed in ‘achieved the guidelines or not’) were examined by entering the cross-product term “change in the framework component × gender” and “change in framework component × baseline PA” in the regression models. Measures of change between the two time points in the implementation scores of the five framework components and in PA were calculated by subtracting the measures at time point 2 from the measures at time point 1. The changes in the implementation scores of the five framework components were recoded into a dummy variable: increased or decreased. All analyses were controlled for two proxy measures of individual SES (educational attainment of mother and father). Furthermore, the analyses were controlled for the type of monitor used by entering a variable “type of monitor (accelerometer/pedometer)” in the regression models. P-values of 0.05 were considered significant.

## Results

### Sample characteristics

In total, for 420 children data were available for both the first and second phase of the study. The sample consisted of 208 girls (49.5%) and 212 boys (50.5%). Mean age at baseline was 11.1 ± 0.5 years and at follow-up 13.4 ± 0.6 years. Approximately half of the children’s parents were highly educated, as 56.3% of the mothers and 47.6% of the fathers attained college or university.

### Changes in physical activity

Table 2 summarizes the results of the Cross-Classified Multilevel Regression Models conducted to investigate longitudinal changes (transition primary school (T1) - secondary school (T2)) in PA. The regression models revealed a significant increase in self-reported active transport to and from school (p < 0.001), a decrease in extracurricular PA (p < 0.001) and a decrease in total PA (p < 0.01) from primary to secondary school. The models also indicated that accelerometer determined minutes of weekday MVPA increased whereas the accelerometer/pedometer determined weekday steps counts showed no significant change from primary to secondary school.

**Table 2:** Longitudinal changes in physical activity during the transition from primary to secondary school

	**T1**	**T2**	**ß**
***Self-reported PA***			
**Active transport to and from school (mean min/day)**	11.35 (13.35)	17.23 (17.83)	5.84 (1.16)***
**Extracurricular PA (mean min/day)**	23.12 (18.67)	10.72 (15.09)	−10.52 (1.26)***
**Total PA level (mean min/day)**	80.62 (41.62)	69.49 (40.42)	−8.90 (2.59)**
***Pedometer/acceleromete determined PA***			
**Pedometer/accelerometer weekday steps (mean steps/day)**	11 242.36 (3548.01)	10 940.92 (3729.48)	−423.98 (255.43)
**Accelerometer weekday MVPA (mean min/day)**^**§**^	27.69 (19.15)	31.74 (23.97)	4.85 (2.42)*

***p < 0.001, **p < 0.01, *p < 0.05.

Physical activity (PA).

Moderate to vigorous physical activity (MVPA).

T1 = first phase, baseline primary school measurements.

T2 = second phase, follow-up secondary school measurements.

^**§**^Data collected in subsample.

Entering the cross-product term “time point × baseline PA” in the regression models revealed that the change in pedometer/accelerometer determined weekday step counts from primary to secondary school is dependent on the level of PA in primary school (Table 3). For children not achieving the PA guidelines in primary school, the mean number of steps on weekdays was low in primary and secondary school and almost no change from primary to secondary school could be observed, while for children achieving the PA guideline at baseline, the mean number of steps in primary school was high but showed a steep decrease from primary to secondary school. For the accelerometer determined MVPA, the self-reported active transport to and from school, extracurricular PA and total PA, no significant “time point × baseline PA” interaction was found (Table 3).

**Table 3:** Moderating effect of gender and the level of physical activity in primary school (expressed in achieved the physical activity guideline or not) on the longitudinal changes in physical activity during the transition from primary to secondary school

**Moderating effect of gender**	**T1 – boys**	**T1 – girls**	**T2 – boys**	**T2 – girls**	**time point × gender (boys/girls)**
					**(ß)**
***Self-reported PA***					
**Active transport to and from school (mean min/day)**	12.62 (14.23)	10.08 (12.29)	18.79 (18.31)	15.65 (17.22)	1.26 (2.33)
**Extracurricular PA (mean min/day)**	27.61 (18.38)	18.52 (17.87)	15.15 (16.84)	6.13 (11.35)	−1.17 (2.53)
**Total PA level (mean min/day)**	92.16 (42.70)	68.84 (37.04)	81.47 (44.67)	57.19 (31.16)	5.03 (5.16)
***Pedometer/accelerometer determined PA***					
**Pedometer/accelerometer weekday steps (mean steps/day)**	12214.93 (3586.93)	10259.82 (3231.38)	12075.09 (3991.01)	9800.48 (3057.36)	199.71 (512.31)
**Accelerometer weekday MVPA (mean min/day)**	34.55 (20.60)	19.78 (13.65)	40.79 (26.57)	21.59 (15.40)	6.16 (4.89)
**Moderating effect of physical activity in primary school**	**T1 – A**	**T1 – B**	**T2 – A**	**T2 – B**	**time point × baseline physical activity (A/B)**
					**(ß)**
***Self-reported PA***					
**Active transport to and from school (mean min/day)**	13.19 (13.65)	10.49 (13.29)	18.22 (18.30)	16.99 (17.58)	−2.77 (2.51)
**PA (mean min/day)**	24.28 (19.06)	22.99 (18.53)	13.16 (15.44)	9.57 (14.76)	−0.28 (2.69)
**Total PA level (mean min/day)**	90.72 (44.89)	76.12(39.90)	78.71 (42.82)	65.35 (38.76)	−7.70 (5.49)
***Pedometer/accelerometer determined PA***					
**Pedometer/accelerometer weekday steps (mean steps/day)**	14811.25 (2817.57)	9478.01(2323.06)	12265.14 (4110.56)	10226.23 (3258.95)	−3498.75 (515.77)***
**Accelerometer weekday MVPA (mean min/day)**	50.67 (20.60)	23.39 (15.51)	48.26 (32.02)	28.52 (20.97)	−6.98 (6.21)

***p < 0.001.

Physical activity (PA).

Moderate to vigorous physical activity (MVPA).

T1 = first phase, baseline primary school measurements.

T2 = second phase, follow-up secondary school measurements.

A = achieved the physical activity guideline in primary school.

B = did not achieve de physical activity guideline in primary school.

Entering the cross-product term “time point × gender” in the regression models revealed that the changes in accelerometer determined MVPA and step counts on weekdays, self-reported active transport to and from school, extracurricular PA and total PA, are not dependent of gender (Table 3).

### Differences in the implementation scores of the five framework components

Table 4 summarizes the results of the Cross-Classified Multilevel Regression Models conducted to investigate differences in the implementation scores of the five framework components concerning extracurricular PA promotion, between primary and secondary schools. These models revealed a significantly higher implementation score for after-school sports and PA (p < 0.001) in primary schools than in secondary schools (score primary school: 3.39 (0.66), score secondary school: 3.28 (0.71). In contrast, the implementation scores of sports and PA during lunch break (p < 0.001, score primary school: 2.65 (0.88), score secondary school: 3.32 (0.86)), active schoolyards and playgrounds (p < 0.001, score primary school: 4.45 (1.88), score secondary school: 5.05 (2.77)) and health education policy (p < 0.001, score primary school: 3.05 (0.76), score secondary school: 3.72 (0.58)) were higher in secondary schools than in primary schools. The implementation score of active commuting to school showed no significant difference between primary and secondary schools (score primary school: 4.09 (0.51), score secondary school: 4.09 (0.38)).

**Table 4:** Longitudinal differences in the implementation scores of the five framework components concerning extracurricular physical activity promotion on schools

	**T1**	**min - max score**	**T2**	**min - max score**	**ß**
**Active commuting to school**	4.09 (0.51)	3.0 - 5.0	4.09 (0.38)	3.2 – 4.9	−0.03 (0.02)
**Sports and PA after school**	3.39 (0.66)	2.0 - 4.6	3.28 (0.71)	1.3 – 4.6	−0.17 (0.04)***
**Sports and PA during lunch break**	2.65 (0.88)	1.4 – 4.4	3.32 (0.86)	1.3 -4.5	0.66 (0.04)***
**Active schoolyards or playgrounds**	4.45 (1.88)	0.0 - 8.0	5.05 (2.77)	0.0 – 10.0	0.42 (0.12)***
**Health education policy**	3.05 (0.76)	2.0 - 5.0	3.72 (0.58)	2.0 – 5.0	0.63 (0.03)***

***p < 0.001.

Physical activity (PA).

T1 = first phase, baseline primary school measurements.

T2 = second phase, follow-up secondary school measurements.

### Associations between changes in the implementation scores of the five active school strategies and changes in physical activity

Table 5 summarizes the results of the Cross-Classified Multilevel Regression Models conducted to investigate if changes in the five framework components concerning extracurricular PA promotion can predict changes in PA. These models revealed that change in the component active schoolyards and playgrounds, is a significant predictor (p < 0.01) of change in the pedometer/accelerometer determined weekday step counts: an increase in the score of active schoolyards and playgrounds is associated with an increase in the weekday steps counts.

**Table 5:** Associations between changes in the implementation scores of the five framework components associated with extracurricular physical activity promotion on schools and changes in physical activity

	**ß**	**ß**	**ß**
	**Active commuting to school**	**Active commuting to school X gender**	**Active commuting to school X T1 PA**
***Self-reported PA***			
**Active transport to and from school (mean min/day)**	−3.722 (2.632)	−3.921 (5.175)	3.88 (5.51)
**Total PA (mean min/day)**	−0.467 (5.746)	−14.087 (11.300)	−27.64 (11.76)*
***Pedometer/accelerometer determined PA***			
**Pedometer/accelerometer weekday steps (mean steps/day)**	459.398 (583.017)	−2081.135 (1135.249) (0.067)	53.58 (1077.28)
**Accelerometer weekday MVPA (mean min/day)**	3.239 (3.251) *(n = 89)*	−3.455 (6.581)	−19.03 (8.36)*
	**Sports and physical activity after school**	**Sports and physical activity after school X gender**	**Sports and physical activity after school X T1 PA**
***Self-reported PA***			
**Extracurricular PA (mean min/day)**	0.769 (3.288)	−1.648 (5.054)	−4.21 (5.57)
**Total PA (mean min/day)**	−7.015 (5.750)	−3.642 (10.996) *(n = 275)*	4.44 (11.86)
***Pedometer/accelerometer determined PA***			
**Pedometer/accelerometer weekday steps (mean steps/day)**	−313.105 (597.789)	818.353 (1109.220) *(n = 261)*	−1485.70 (1042.65)
**Accelerometer weekday MVPA (mean min/day)**	−4.837 (3.294) *(n = 96)*	−1.112 (6.531) *(n = 96)*	−7.14 (8.16)
	**Sports and physical activity during lunch break**	**Sports and physical activity during lunch break X gender**	**Sports and physical activity during lunch break X T1 PA**
***Self-reported PA***			
**Extracurricular PA (mean min/day)**	0.652 (3.248)	7.099 (5.344)	3.29 (5.95)
**Total PA (mean min/day)**	8.060 (6.225)	−11.067 (11.746)	−1.70 (13.45)
***Pedometer/accelerometer determined PA***			
**Pedometer/accelerometer weekday steps (mean steps/day)**	349.558 (621.506)	770.336 (1171.880)	−188.36 (1158.95)
**Accelerometer weekday MVPA (mean min/day)**	−1.086 (3.487) *(n = 98)*	−3.969 (6.857)	8.10 (10.43)
	**Active schoolyards or playgrounds**	**Active schoolyards or playgrounds X gender**	**Active schoolyards or playgrounds X T1 PA**
***Self-reported PA***			
**Extracurricular PA (mean min/day)**	−2.015 (3.116)	8.898 (4.767) (0.061)	4.33 (5.32)
**Total PA (mean min/day)**	1.903 (5.732)	25.254 (10.524)*	2.07 ( 12.09)
***Pedometer/accelerometer determined PA***			
**Pedometer/accelerometer weekday steps (mean steps/day)**	1496.011 (540.137)**	1185.26 (1043.833)	−338.73 (1034.24)
**Accelerometer weekday MVPA (mean min/day)**	1.019 (3.230) *(n = 97)*	1.050 (6.151)	−10.34 (8.30)
	**Health education policy**	**Health education policy X gender**	**Health education policy X T1 PA**
***Self-reported PA***			
**Extracurricular PA (mean min/day)**	6.816 (3.380)*	1.713 (5.669)	4.09 (6.38)
**Total PA (mean min/day)**	4.193 (6.417)	−21.087 (12.598)	7.26 (14.29)
***Pedometer/accelerometer determined PA***			
** Pedometer/accelerometer weekday steps (mean steps/day)**	773.780 (684.777)	−1213.547 (1340.886)	−1014.20 (1251.11)
** Accelerometer weekday MVPA (mean min/day)**	0.033 (3.656) *(n = 98)*	−10.613 (7.316)	−11.74 (9.35) (n = 97)

**p < 0.01, * p < 0.05.

Physical activity (PA).

Moderate to vigorous physical activity (MVPA).

Furthermore, a positive change in health education policy significantly predicts (p < 0.05) an increase in extracurricular PA.

**Figure 1:**
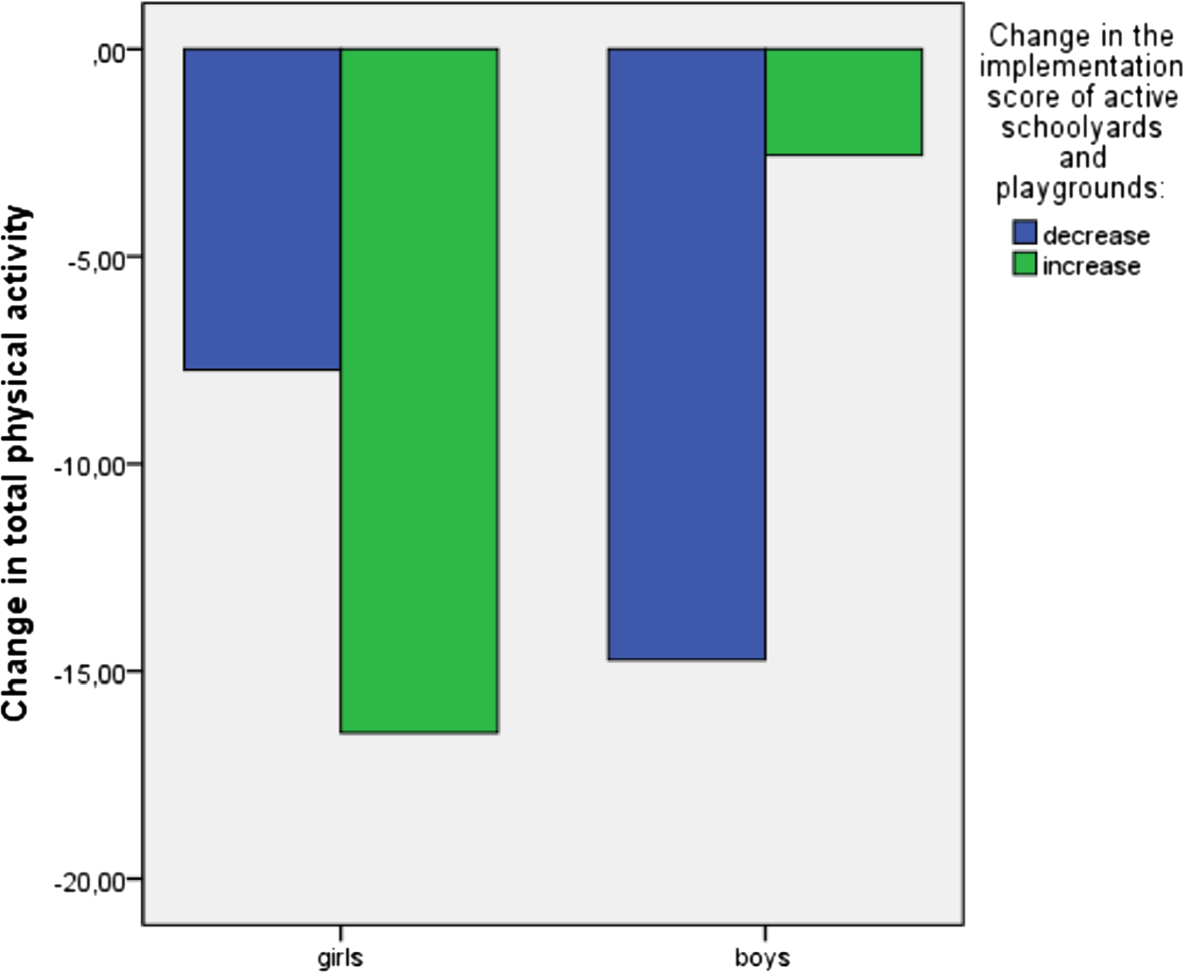
Interaction “change in score of active schoolyards and playgrounds × gender” for total PA.

Entering the cross-product term “change in the framework component × gender” in the regression models revealed that gender is a significant moderator (p < 0.05) of the association of change in active schoolyards and playgrounds with change in total PA (Table 5). Figure 1 shows that among boys, total PA showed a larger decrease when the score on active schoolyards and playgrounds decreased from primary to secondary school. In contrast, among girls, total PA showed a smaller decrease when the score on active schoolyards and playgrounds decreased from primary to secondary school. No other cross-product terms were found to be significant (Table 5).

Entering the cross-product term “change in the framework component x level of PA” in the regression models revealed that the level of PA in primary school (expressed in ‘achieved the guidelines or not’) is a significant moderator (p < 0.05) of the association between change in the score of active commuting to school and change in total PA (Table 5).

**Figure 2:**
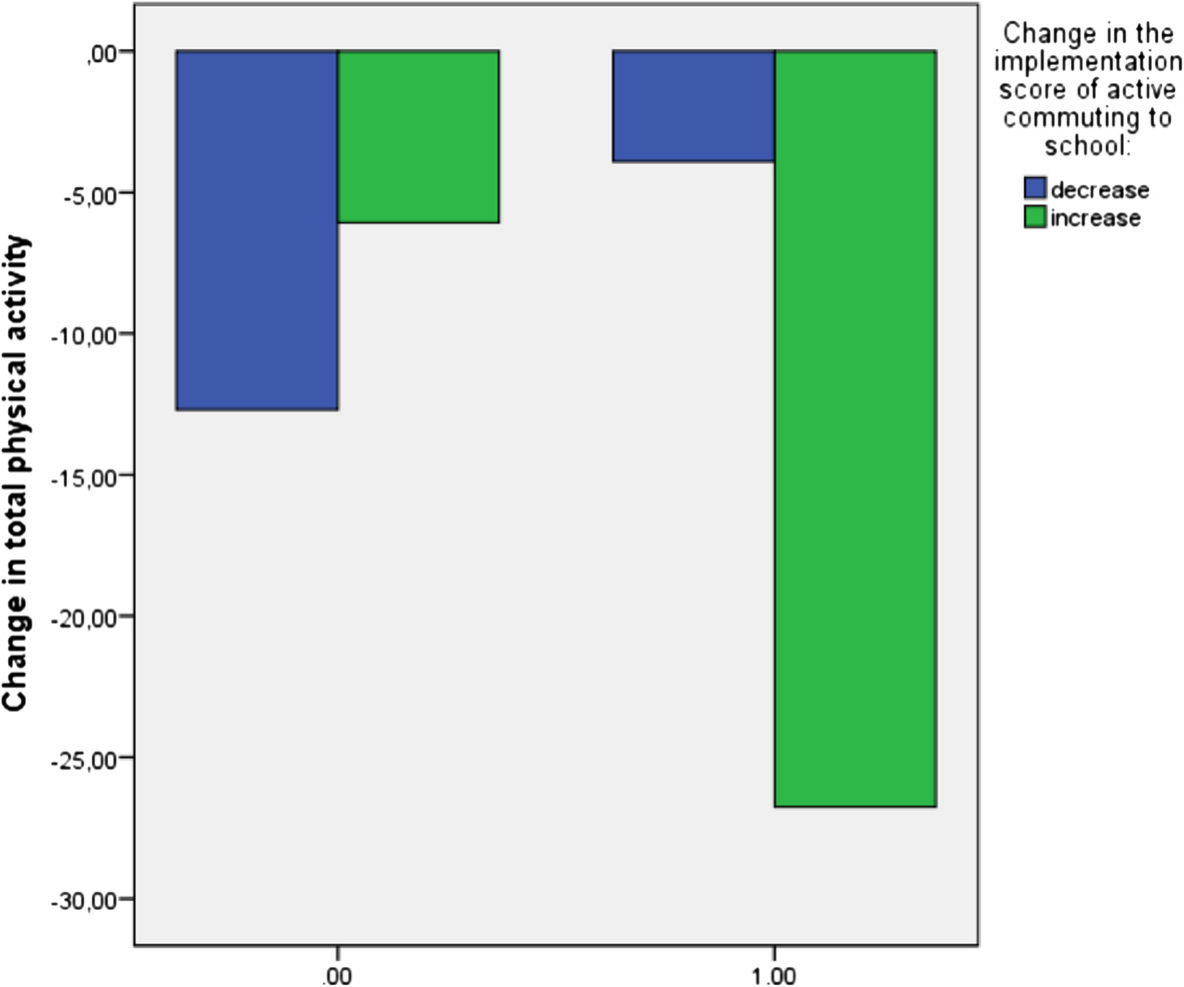
Interaction “change in the score of active commuting to school × baseline physical activity (0 = did not achieve the guidelines, 1 = achieved the guidelines)” for total physical activity.

Among children who did not achieve the PA guideline in primary school, total PA showed a larger decrease when the score of active commuting to school decreased from primary to secondary school. In contrast, among children who achieved the PA guideline in primary school, total PA showed a smaller decrease when the score of active commuting to school decreased from primary to secondary school (Figure 2).

**Figure 3:**
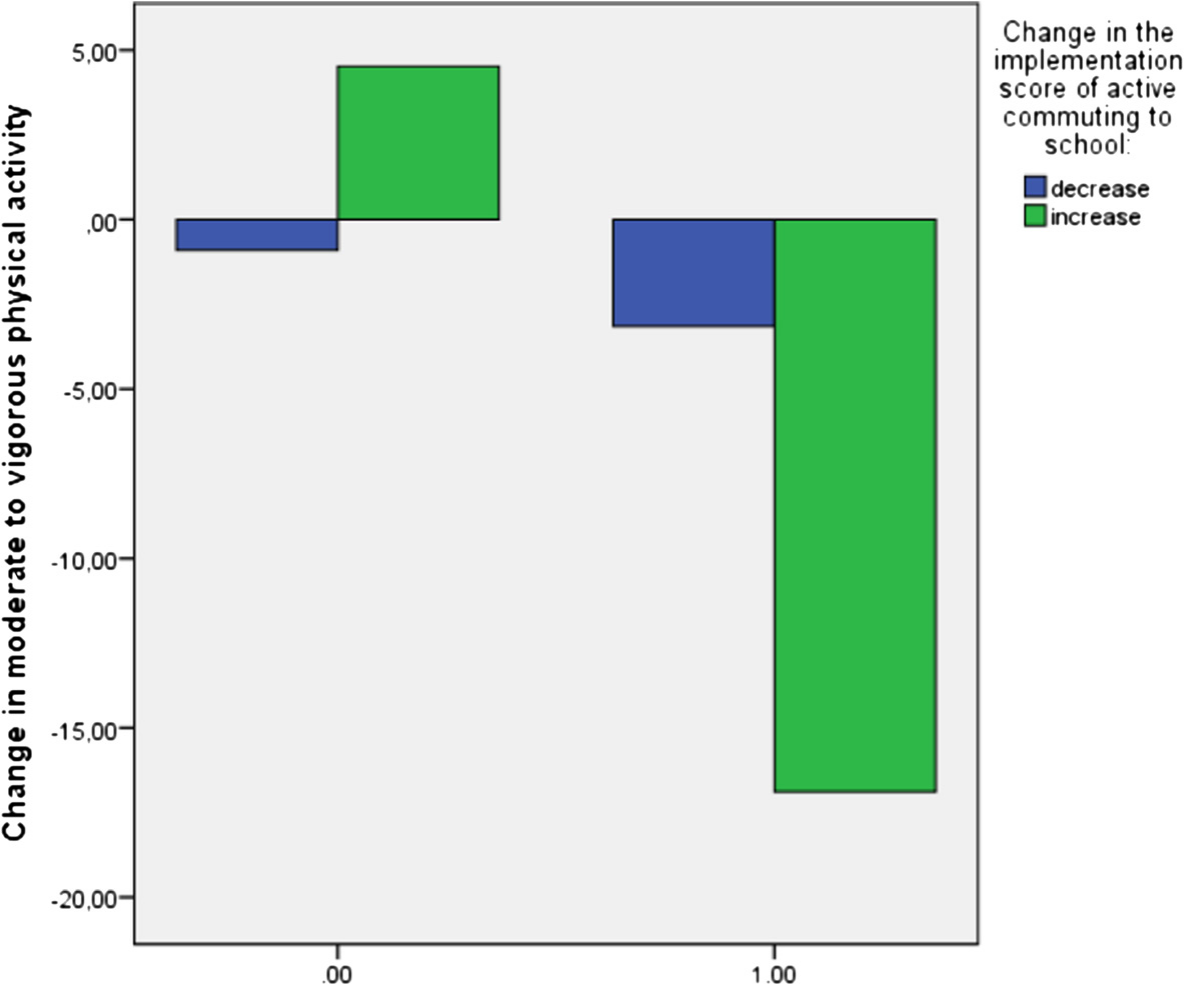
Interaction “change in the score of active commuting to school × baseline physical activity (0 = did not achieve the guidelines, 1 = achieved the guidelines)” for accelerometer determined MVPA.

A comparable moderating effect of the PA level in primary school was found for the association between change in active commuting to school and change in accelerometer determined MVPA (Table 5). Among children who did not achieve the PA guideline in primary school, accelerometer determined MVPA showed a decrease when the score of active commuting to school decreased from primary to secondary school while accelerometer determined MVPA showed an increase when the score of active commuting to school increased. On the other hand, among children who achieved the PA guideline in primary school, accelerometer determined MVPA showed a smaller decrease when the score of active commuting to school decreased from primary to secondary school than when the score of active commuting to school increased (Figure 3).

## Discussion

The results of this study confirm that the transition from primary to secondary school is characterized by a change in PA. In line with the literature [8,11,12] the changes in PA seem to be dependent on the context of PA. Comparable to the results of the study of Cardon and colleagues [12], the data presented here indicated an increase in the self-reported number of minute’s active transport to and from school after the transition from primary to secondary school. Furthermore, a decline was found in self-reported extracurricular PA and total PA. This is in line with the results of the study of Niven and colleagues [8].

Accelerometer determined weekday MVPA increased while for pedometer/accelerometer determined weekday step counts a large decline was observed among those who achieved the PA guidelines in primary school. Among those who did not achieve the guidelines in primary school weekday step counts remained nearly stable. This result underscores the fact that the start level of PA predicts the change in weekday step counts after the transition from primary to secondary school. To our knowledge, this was the first study to investigate changes in weekday PA determined by accelerometers or pedometers. More research is clearly needed to investigate this in more detail. A somewhat surprising result was that no differences between boys and girls were noted in the changes in PA. In two previous longitudinal studies the changes in PA were more apparent in boys than in girls [9,11]. Despite the fact that in the present study no gender differences were found in the change in PA, the results of previous studies underscore the fact that future research still should be encouraged to investigate differences between boys and girls.

The implementation scores of sports and PA during lunch break, active schoolyards and playgrounds and health education policy were higher (increase > 0.50) in secondary schools than in primary schools. Although the change in the score of sports and PA after school was small, the score was significantly lower in secondary compared to primary schools (decrease < 0.50) and the score of active commuting to school showed no difference. In general, secondary schools seem more likely to foster strategies to promote PA during school hours than primary schools who seem more likely to foster strategies to promote PA after school. Primarily, secondary schools are overall larger and have therefore more available space and facilities than primary schools. This may explain the higher implementation score of active schoolyards and playgrounds. Secondly, the availability of space and facilities may in turn be conducive to organize sports and physical activity during lunch break. This may explain the higher implementation score on this component. Third, larger schools typically have more teachers. Consequently, teachers following refresher courses on sports and PA during school hours can be replaced more easily. This ultimately may encourage following refresher courses on sports and PA. Following this reasoning, secondary schools may have a higher score on the item concerning training for teachers that contributes to the implementation score of health education policy. Further, a higher implementation score on the component sports and PA during lunch break and the component active schoolyards or playgrounds may affect the subjective norm with regard to the importance of sport and PA for the school. The subjective norm with regard to the importance of sport and PA for the school is one of the items that determined the component health education policy. And as last, the implementation score of health education policy is also determined by how pupils are involved in the decision making about sports and physical activity. The cognitive abilities of secondary school children may be a plausible explanation for a higher involvement in the decision making process. A possible reason for the higher score of sports and PA after school in primary schools can be that in primary schools after school PA programs are seen as a solution for children who are not allowed to go home after school without supervision. Sports and PA programs after school are then used as a form of after-school care.

An earlier study investigating the implementation of the different components of the PA promotion framework in Flemish primary and secondary schools did not find a difference in the implementation score of the different components of the framework to promote extracurricular PA [19]. However, since the implementation score in the study of Cardon and colleagues is an overall score, including the different implementation scores on the different components of the PA promotion framework and in the present study separate scores were conducted for the five components comparison of the study results is difficult.

To our knowledge this was the first longitudinal study that investigated if changes in the school environment can predict changes in self-reported and pedometer/accelerometer determined PA in different contexts. It was promising to find that changes in three components of a framework that was developed to promote extracurricular PA programs were associated with changes in PA.

An increase in the implementation score of health education policy was found to predict an increase in self-reported extracurricular PA independent of gender or the level of PA at baseline. This result underscores the need to further endeavor for a sport and PA minded school-atmosphere. Efforts to change the overall school spirit, the involvement of students in the decision making process about sports and PA and the support teachers receive to have training on sports and PA may be promising.

A positive association was found between the implementation score of active schoolyards and playgrounds with pedometer/accelerometer determined weekday step counts. Furthermore, among boys, total PA decreased less when the implementation score of active schoolyards and playgrounds increased from primary to secondary school while among girls the decrease in total PA was larger when the implementation score of active schoolyards and playgrounds increased from primary to secondary school. Based on this result it seems that the availability of facilities and equipment seems to be important for boys’ total PA but not for girls. This finding is in accordance to the conclusions of several observational studies concluding that PA facilities in school settings are predominantly used by boys while girls less claim the activity facilities [45,46]. A possible explanation can be that the facilities available are more interesting for activities preferred by boys than by girls. Furthermore, for girls recess and lunch break are considered to be an opportunity to socialize with friends [47].

Active commuting to school was the third framework component that was of importance. The association found between the change in the implementation score of active commuting to school and both self-reported total PA and accelerometer determined MVPA was dependent on the level of PA at baseline. For both total PA and MVPA, an increase in the implementation score of active commuting to school was found to have a more positive effect on children who did not achieve the guidelines in primary school compared with those who did achieve the guidelines in primary school. Children who were not sufficiently active in primary school seem more impressible to the promotion of active commuting to school, the availability of facilities for active commuting and safety issues related to active commuting, than children who did achieve the guidelines in primary school. It is possible that children who were not sufficiently active in primary school are not interested in sports activities but are more receptive for physical activities that are not sports activities. Surprisingly, this component does not directly predicts the change in active commuting, A possible explanation for these findings can be that schools that promote active commuting to school, provide facilities for active commuting, are situated in a safe neighborhood and consequently score higher on the implementation score of active commuting, score also higher on other factors. For example, it is possible that schools with higher scores on the implementation score of active commuting score also higher on the implementation score of active schoolyards and playgrounds This may have had an influence influences on the results.

Two components were no predictors of changes in PA: sports and PA after school and sports during lunch break. This finding is somewhat surprising. Earlier studies showed that factors like access to activity facilities [48], access of equipment to be physically active [49-52], provision of after-school PA [53] and recess PA programs [54] have been associated with participation in extracurricular PA. These factors were used in this study to determine the implementation scores of sports and PA after school and sports during lunch break. However, there is limited data available on adolescents. It is possible that among primary school children but not among older, secondary school children, these components are of importance and can contribute to PA. Consequently, there is a need for additional research to investigate this in more detail.

### Limitations and strengths

The results of this study need to be interpreted in light of some limitations. First, this study has been conducted in a Belgian sample and focuses on a conceptual framework for PA programs that has been developed for schools in Flanders (Belgium). Although some lessons could be learned from the results and conclusions of this study, the findings are not fully generalisable to other countries or continents. Second, step-counts were determined using the Yamax Digi-Walker CW701 and the GT1M accelerometer. Although the step counts measured by the Yamax Digi-walker CW-701 have been shown to be highly correlated with the step counts of the GT1M accelerometer, the overall agreement between the step counts of both monitors is rather low [29]. To overcome this problem, all analyses were controlled for the type of monitor used. Third, the choice was made to determine the level of physical activity in primary school using data that were available for every participant. Because of the limited availability of accelerometers, accelerometers were only used in a subsample. Step-count data was available for every participant and therefore used to determine the level of PA in primary school. Fourth, school SES may have had an impact on the resources of the school and the attention of the school to PA-focused initiatives. Unfortunately, no information was available concerning school SES. Consequently, it was not possible to control the analyses for school SES.

A first strength of the present study is the longitudinal design of the study. Secondly, clustering in primary and secondary schools was taken into account by using cross-classified multilevel analyses.

## Conclusions

This study provides more insight into the changes in PA during the transition from primary to secondary school, the differences in school environmental characteristics with regard to the five components of the Flemish framework to promote extracurricular PA, and the effect of changes in these school environmental characteristics on changes in self-reported and pedometer/accelerometer determined PA. Based on the results of this study it was possible to conclude that the changes found in PA were dependent of the context of PA. Furthermore, secondary schools seem more likely to foster in-school strategies (sports and PA during lunch break, active schoolyards and playgrounds and health education policy) than primary schools while primary schools are more likely to foster sports and PA after school than secondary schools. As last, the implementation of three components of the framework developed to promote extracurricular PA were found to be important to predict changes in PA during the transition from primary to secondary school: active commuting to school, active schoolyards and playgrounds and health education policy. As these components can contribute to higher levels of PA, efforts are needed to extent the implementation of these components in all primary and secondary schools. Moreover, components need to be implemented in school settings as early as possible to encounter the changes in PA in a positive manner during the transition from primary to secondary school. However, the contribution of other social and physical environmental factors to the changes in PA need to be further explored.

## Competing interests

The authors declare that they have no competing interests.

## Authors’ contributions

All authors read and approved the final version of the manuscript. FDM coordinated the data collection, assisted in the recruitment of the participants, conducted the statistical analyses and drafted the manuscript. GC, DVD, IDB and BD participated in the interpretation of the data, revised the draft versions of the manuscript and provided critical comments during the process.

## Pre-publication history

The pre-publication history for this paper can be accessed here:

http://www.biomedcentral.com/1471-2458/14/261/prepub
